# Response biomarkers: re-envisioning the approach to tailoring drug therapy for cancer

**DOI:** 10.1186/s12885-016-2886-9

**Published:** 2016-11-05

**Authors:** Shahil Amin, Oliver F. Bathe

**Affiliations:** 1 Cumming School of Medicine, Faculty of Graduate Studies, University of Calgary, Calgary, Canada; 2 Department of Surgery, University of Calgary, Calgary, Canada; 3 Department of Oncology, University of Calgary, Calgary, Canada; 4 University of Calgary, Arnie Charbonneau Cancer Research Institute, Health Research Innovation Centre, 2AA-07, 3280 Hospital Drive NW, Calgary, AB T2N 4Z6 Canada; 5 Tom Baker Cancer Center, 1131 29th Street NW, Calgary, AB T2N 4 N2 Canada

**Keywords:** Response Biomarker, Predictive biomarker, RECIST, Assessing response, Adaptive biomarker, Systemic therapy, Cancer

## Abstract

**Background:**

The rapidly expanding arsenal of chemotherapeutic agents approved in the past 5 years represents significant progress in the field. However, this poses a challenge for oncologists to choose which drug or combination of drugs is best for any individual. Because only a fraction of patients respond to any drug, efforts have been made to devise strategies to personalize care. The majority of efforts have involved development of predictive biomarkers. While there are notable successes, there are no predictive biomarkers for most drugs. Moreover, predictive biomarkers enrich the cohort of individuals likely to benefit; they do not guarantee benefit.

**Main text:**

There is a need to devise alternate strategies to tailor cancer care. One alternative approach is to enhance the current adaptive approach, which involves administration of a drug and cessation of treatment once progression is documented. This currently involves radiographic tests for the most part, which are expensive, inconvenient and imperfect in their ability to categorize patients who are and are not benefiting from treatment. A biomarker approach to categorizing response may have advantages.

**Conclusion:**

Herein, we discuss the state of the art on treatment response assessment. While the most mature technologies for response assessment involve radiographic tests such as CT and PET, reports are emerging on biomarkers used to monitor therapeutic efficacy. Potentially, response biomarkers represent a less expensive and more convenient means of monitoring therapy, although an ideal response biomarker has not yet been described. A framework for future response biomarker discovery is described.

## Background

For many solid tumors, the therapeutic armamentarium is rapidly expanding, particularly with advances in molecularly-targeted drugs. But only a fraction of patients are responsive to any antineoplastic drug, and there is a need to better tailor therapy for any individual. The present approach to the palliative management of solid tumors involves administering a drug (or combination of drugs) that the oncologist speculates will be effective in a given tumor type. Following a significant exposure to chemotherapy (typically over several months), the oncologist estimates response radiographically. However, the radiographic features of a response to chemotherapy are not always obvious. Moreover, if disease progression occurs while on chemotherapy, the patient has had to suffer any toxicities related to the drugs; and the patient’s condition may have deteriorated (due to disease progression, as well as toxicities). This could interfere with administration of subsequent lines of chemotherapy. Meanwhile, the payer is saddled with the costs of an ineffective therapy.

There is little argument that oncologic care must be personalized. Biomarkers represent one strategy to tailor therapy. However, the vast majority of our efforts have focused on development of prognostic and predictive biomarkers, which has had limited success. Response biomarkers have not been thoroughly explored. The purpose of this review is to discuss the potential advantages of response biomarkers, and to envisage how a better response biomarker might transform clinical practice as well as drug development.

### Increasing complexity of the therapeutic landscape: the impending crisis

In recent decades, chemotherapeutic agents used in clinical practice consisted mainly of cytotoxic drugs. The stochastic increase in response rates in some tumor types resulted mostly from drugs used in combination, at the cost of some increase in toxicity. More recently, there has been a rapid proliferation of agents that specifically target an ever expanding array of molecules. In general, these molecularly targeted agents are cytostatic, making it more difficult to assess their contribution to the health of the patient.

**Fig. 1:**
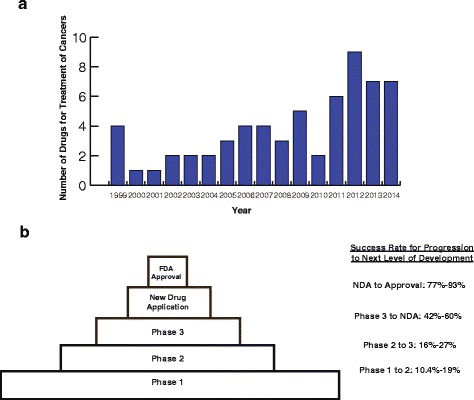
The drug development pipeline for cancer. **a** Number of drugs approved each year by the FDA for the treatment of cancer, since 1999. Figure is derived from the annual briefs on New Molecular Entity and New biologic Approvals [74]. **b** Probability of success in advancing a proposed therapeutic compound from phase 1 clinical trials to FDA approval. Data are derived from Hay et al. [75]

The rate of FDA drug approval for treatment of cancers has been accelerating (Fig. 1a). Therefore, for the practicing oncologist, the choice of which agent (s) to administer to any individual is becoming more complex. At the same time, oncologists are limited to drugs approved by their formulary. Cost and evidence of effectiveness from large clinical trials affect the availability of drugs in the formulary, perhaps restricting access to potentially effective drugs in an individual.

The drug development pipeline is sizeable. As of the time of this writing, it is estimated that 320 drugs are in phase I and II stages of development [1]. Given the finite patient resources and financial constraints of industry and clinical trial groups, only a small proportion of these drugs will ever reach phase III trials (Fig. 1b). The cost of developing a drug is estimated to be a staggering $1.3 billion [2]. Even those drugs tested in phase III trials may never be adopted into clinical practice because they do not increase survival in the *aggregate* patient population, or because the magnitude of their benefit to the aggregate is insufficient to warrant the costs. This bottleneck has some important implications. First, a number of potentially useful drugs may remain untested in phase III trials because so many drugs with a positive phase II signal are competing for inclusion in larger trials. Second, drugs that are useful to individuals may not be approved because of insufficient effect on the study population as a whole.

Clearly, a more efficient approach is required to develop and test drugs, to determine which drug (s) benefit an individual, and to ensure that drugs that benefit individuals (but perhaps not the aggregate) are available.

### The problem with predictive biomarkers

Most systemic agents or drug combinations used for solid tumors only benefit a fraction of individuals. This is readily observable whenever progression-free survival (PFS) is illustrated for any drug trial. Therefore, given the toxicity of these agents as well as their cost, there is a need to identify individuals who will benefit. Presently, the dominant approach to personalizing therapy involves the development of predictive biomarkers. While a few predictive biomarkers have entered clinical practice (including KRAS mutation status, Her-2 expression, and estrogen receptor expression), development of predictive biomarkers is associated with a number of challenges.

Most importantly, predictive biomarkers are typically specific to a particular agent; they reflect the presence of the molecular derangements necessary for any drug to exert its biological effect and the absence of mechanisms of drug resistance. Therefore, for any new drug that becomes available, new avenues of research must be developed to identify and validate predictive biomarkers for that new drug. As chemotherapeutic options become more numerous, diagnostic laboratories will require competence in more assays. The whole process of developing predictive biomarkers is therefore expensive and time consuming.

Predictive biomarkers also do not guarantee benefit. Rather, they are helpful in excluding patients from getting a drug that will *not* benefit. This is illustrated in the case of epidermal growth factor receptor (EGFR) in colorectal cancer. A KRAS mutation predicts that an EGFR inhibitor will not be beneficial; only about 1 % respond to cetuximab. On the other hand, only 12.8 % of individuals with KRAS wildtype have a measurable response, and less than 60 % have a longer progression free survival (PFS) than the median survival of patients treated with best supportive care [3]. Similarly, the absence of estrogen receptor (ER) in breast cancer indicates resistance to hormonal therapy, but only 50–75 % of ER positive tumors respond to various hormone manipulations [4, 5]. Predictive markers are therefore far from predictive.

Finally, there is the problem of defining a predictive biomarker. Predictive biomarkers are defined and validated in randomized controlled trials in which a treatment is not administered to a control group. In the absence of a non-treatment group, it is difficult to discriminate whether a biomarker that categorizes patient survival is predictive or prognostic (reflecting biological subsets). Prognostic biomarkers do not aid in making go/no-go treatment decisions.

### Response as an endpoint for drug development and approval

Generally, for a cancer drug to be approved and introduced to clinical practice, it must have an impact on survival. However, an aggregate survival benefit may be too stringent a criterion, particularly in the advent of targeted therapy, where ever-smaller chemosensitive subgroups have not been fully defined. Drugs that benefit only the few will not measurably impact aggregate survival unless there is some way to enrich a study cohort with chemosensitive participants.

There have been exceptions where drugs have been approved without demonstrable survival benefit. One example is the approval of gemcitabine for unresectable pancreatic cancer, based on an improvement in median survival from 4.2 months to 5.7 months [6]. While this was not a great improvement on the surface, one year survival increased from 2 to 18 %. Objective response rate was very low (5.4 %) [6], but there was an improvement in “clinical benefit response”, which reflects improvements in disease-related symptoms. Importantly, there were no good treatment alternatives. Gefitinib failed to demonstrate a survival benefit in large trials on non-small cell lung cancer [7, 8]. However, it was approved based on a surrogate endpoint for clinical efficacy (response rate, which was about 10 %). There is therefore some precedent for approval of drugs based on benefits to the few and based on response.

In a wide variety of circumstances, progression free survival (PFS) is considered a good surrogate endpoint [9–11]. In those conditions, clinical trials could be done more economically and more quickly than trials where overall survival is the primary endpoint. On the other hand, the magnitude of treatment effects on PFS is known to be higher than the effects on OS [12]. Therefore, to some degree, as a community, we will need to assign some value to achieving a progression free interval.

Similarly, objective response (ie: reduction in tumor size or attenuation; metabolic response) is associated with a survival benefit in some studies [9, 13, 14]. Indeed, if this were consistently the case, then early phase trials could be designed using response as a primary endpoint, which would dramatically accelerate drug development and maybe even result in a more immediate refinement of the target population for later stage trials.

There are several problems related to using response as a clinical trial endpoint at this time. First, the relationship between response and survival is indirect: it is not clear whether improved survivals are due to the response per se or because of generally favorable tumor biology. The role of biology is apparent in a surgical series of colorectal liver metastases reported by Adam et al., who observed that progression following neoadjuvant chemotherapy (“bad biology”) was associated with poor survivals after resection [15]. To emphasize this point, Petrelli and coworkers have observed that, in metastatic colorectal cancer, early tumor shrinkage is prognostic but not sufficiently correlated with overall survival to act as a surrogate [13]. Second, the significance of stable disease is not obvious. In some instances, stable disease may represent a response; in others, it may represent indolent tumor biology. Finally, response rate is a function of methodology. Changes in tumor size, attenuation and metabolic activity each reflect different drug effects; depending on the types of drugs used, response rates vary depending on how they are measured. Therefore, work is needed to refine methods of measuring response and to establish the linkage of those refined measures to clinical benefit.

### Current methods of assessing response to therapy

#### Standard radiographic assessment

Presently, response to treatment is assessed radiographically – typically CT scan or MRI. The criteria for response typically utilized for solid tumors treated with cytotoxic agents are the RECIST criteria, based on changes in tumor size [16, 17]. But the RECIST criteria are not well suited for some situations. With some tumor types, including esophagogastric cancers and biliary cancers, tumor extent is difficult to assess radiographically. Cancers that have spread to involve the peritoneum and the pleura are similarly difficult to measure. In these circumstances, RECIST criteria are not helpful for the assessment of a treatment response. Some cytotoxic treatments are not associated with reductions in tumor dimension. For example, hepatocellular carcinoma (HCC) submitted to locoregional treatments such as transarterial chemoembolization and radiofrequency ablation cause a high degree of tumor necrosis, but there is often no accompanying reduction in size [18]. RECIST criteria therefore underestimate therapeutic response rates.

RECIST criteria are similarly problematic for response assessment following administration of targeted agents. These agents are typically cytostatic, not cytotoxic, and changes in the dimensions of the tumors are seen less frequently [19, 20]. Therefore, by RECIST criteria, response is underestimated. In instances of stable disease (by RECIST criteria), it is also difficult to distinguish stability due to therapy versus stability due to indolent tumor biology. To address this problem, Choi and coworkers have described the use of CT to assess for changes in attenuation [20]. Therapy-related reductions in tumor attenuation, which may reflect inhibition of angiogenesis or decreased tumor viability, are reportedly associated with better progression-free survivals for gastrointestinal stromal tumors (GIST), renal cell carcinoma (RCC) and HCC [19–21]. The Choi criteria are therefore considered an important adjunct in response evaluation following systemic treatment with molecular-targeted agents.

Traditional response criteria may not be appropriate for immune interventions, such as immune checkpoint blockade, vaccines and adoptive therapy. As in the new molecularly targeted agents, meaningful responses are often associated with minimal or no reduction in tumor size. Interestingly, in some individuals, progressive disease (as estimated by RECIST criteria) precedes a reduction in tumor dimension [22, 23]. This is not unlike the situation following radiotherapy, where immediate post-radiation changes may invoke an inflammatory response accompanied by an increase in tumor dimension [24]. For this reason, it has been proposed that response after immunotherapy be classified using specialized criteria. Using these criteria, treatments are not discontinued immediately with progressive disease unless progression is sustained and confirmed [25].

#### Positron emission tomography (PET)

Functional imaging techniques have also been used to assess treatment response. The most widely available platform is [18 F] fluorodeoxyglucose (FDG) PET, which reflects the metabolic activity of tumor. A reduction in FDG avidity is observed with effective treatment. This has been used effectively for monitoring response to cytotoxic therapies as well as in targeted therapies [9]. Response can be categorized as soon as 4 weeks after treatment [26]. Generally, metabolic response precedes anatomic response, and metabolic response rate exceeds response rate as determined by RECIST, yet metabolic response still corresponds to improved survival [9].

While FDG-PET is most widely available, other radiotracers have some potential utility. 3′-deoxy-3′-18 F fluorothymidine PET (FLT-PET) has interesting features as a test for assessing response. FLT is taken up by rapidly proliferating cells, and reductions in maximum tumor standardized uptake value (SUVmax) from baseline have been reported within 7 days of starting gefitinib in advanced lung adenocarcinoma patients [27]. Similarly, changes in FLT avidity have been reported as early as a week after chemotherapy for breast cancer. Importantly, FLT-PET can distinguish between a clinical response and stable disease [28]. [18 F] fluorocholine PET (FCH-PET) is based on increased choline uptake by cancer cells because of increased phosphatidylcholine requirements for cell membrane formation in highly proliferative cells [29]. FCH-PET has similarly been used to assess response in patients treated with enzalutamide for metastatic castration-resistant prostate cancer (CRPC). Early FCH-PET predicted progressive disease 3 months before CT in 66 % of patients and was a significant predictor of progression free survival [30].

With the advent of PET, new criteria for response to treatment have been developed, Positron Emission Tomography Response Criteria in Solid Tumors (PERCIST) [31]. The PERCIST criteria enable assessment of response in tumors that may not change in size, but instead have a functional decline, most typically a reduction in glycolysis (as reflected by FDG avidity). Solid tumors invisible on anatomical imaging can therefore be tracked. In a study of patients receiving neoadjuvant chemotherapy for breast cancer, FDG-PET and PERCIST criteria had greater sensitivity, specificity and accuracy in predicting pathologic complete response (70.4, 95.7 and 90.8 %, respectively) compared to RECIST utilizing MRI (45.5, 85.5 and 82.4 % respectively) [32]. In a group of patients with non-small cell lung cancer, PERCIST criteria, but not RECIST criteria, predicted disease free survival [33]. More recently, PERCIST metabolic response was able to predict overall and progression free survival in patients with pancreatic cancer liver metastases treated with ^90^Y-Yttrium microspheres [34].

The use of PERCIST criteria to measure response has some limitations. PET scans are not widely available and repeated studies are expensive to execute. Moreover, in many clinical facilities, PET scans are not implemented in a manner that allows accurate calculation of PERCIST criteria.

#### Other functional imaging modalities

Dynamic contrast enhanced ultrasonography (DCE-US) is an alternative functional imaging technique that enables quantitative assessment of tumor perfusion. It may therefore play a role in assessing the efficacy of antiangiogenic agents. DCE-US peak intensity was shown to be a predictive tool in indicating early response efficacy of sunitinib treated RCC patients 15 days after treatment [35]. In HCC patients, DCE-US has been useful in identifying patients responding to sorafenib [36] and axitinib [37]. Further clinical trials are in progress for evaluating the roles of 3D dynamic contrast enhanced ultrasound imaging contrast enhanced ultrasound, and shear wave elastography.

#### Circulating tumor cells (CTCs)

CTCs can be detected by evaluation of tumor-specific mRNA transcripts by reverse transcription polymerase chain reaction. In general, this approach has been difficult to standardize because of the use of different primers and assay conditions, making it difficult to compare results between labs. Since the introduction of assay systems to enumerate CTCs, a number of studies have demonstrated that higher numbers of CTCs are associated with a worse survival in a variety of tumor types [38–41]. It therefore follows that a treatment-induced reduction of CTCs would reflect treatment efficacy. In metastatic breast cancer patients, a reduction in CTCs after 3–4 weeks of treatment correlates with radiographic response [42]. Also in metastatic breast cancer patients, a longer PFS is seen in patients with <5 CTCs following initiation of systemic therapy [43]. Overall survival is better in metastatic breast cancer and castration resistant prostate cancer (CRPC) patients where there is a treatment-related reduction in the numbers of CTCs [44, 45]. In patients with neuroendocrine tumors receiving various therapies, post-treatment reductions in CTCs exceeding 50 % were associated with improved survivals [46]. Monitoring CTCs during treatment therefore represents an attractive strategy to monitor treatment efficacy. The main problem with this approach is that accurate interpretation is difficult when CTCs are undetectable or at low numbers. Therefore, its implementation in all patients is hindered in that population.

#### Circulating nucleic acids

Circulating tumor DNA (ctDNA) has been measured to predict treatment outcome and assess response to therapy [47–50]. In metastatic colorectal cancer patients treated with first line combinations of oxaliplatin or irinotecan (with or without biological therapy), significant changes in ctDNA were seen as early as 3 days after initiating chemotherapy. The reductions in ctDNA seen by 14–21 days correlated to response (measured by CT using RECIST criteria). In patients who had ≧10 fold reduction in ctDNA levels, 74 % had a measurable response on CT; patients who had reductions in ctDNA of this magnitude had a significant improvement in PFS [51]. In metastatic melanoma patients treated with MAPK inhibitors, measurable responses were accompanied by reductions in ctDNA after 4 – 8 weeks of therapy. Interestingly, in a group of patients treated with immunotherapies (ipilimumab, nivolumab or pembrolizumab), there was no significant reduction in ctDNA. The authors also presented data that suggested this strategy could be used for early detection of acquired resistance [52].

Circulating microRNAs (miRs) have also been used to measure disease burden. Plasma levels of miR-155, 197 and 182 significantly decreased with response to chemotherapy in a small group of lung cancer patients [53]. Serum miR-155 levels were decreased in breast cancer patients after surgery, but there were no definitive data on the effects of chemotherapy on miR-155 levels [54]. Following surgery in colorectal cancer patients, circulating miR-17-3p and miR-92 levels drop [55]. In metastatic colorectal cancer patients treated with XELOX and bevacizumab, miR-126 levels decreased in responders and increased in non-responders [56]. In 23 non-small cell lung cancer patients undergoing combined therapy, increasing levels of miR-19b and decreasing levels of miR-125b were associated with a therapeutic response [57].

Finally, long non-coding RNAs (lncRNA) have also been used to assess response. In a small group of head and neck cancer patients, following chemoradiotherapy, there was a greater reduction in circulating lncRNA GAS5 levels associated with complete response compared to PR/SD. Other lncRNAs did not change with response [58].

#### Circulating tumor markers

Tumor markers that are reliably elevated with disease and that accurately reflect tumor burden may be used to measure response. Unfortunately, those conditions are infrequently met in most instances. Regardless, some studies have shown the utility of using tumor markers to assess response. In patients with HCC treated with sorafenib, survival was improved in individuals with a >20 % decrease in alphafetoprotein [59]. In a cohort of patients with colorectal liver metastases, a reduction of >20 % in carcinoembryonic antigen (CEA) was highly correlated with radiographic response [60]. Moreover, in locally advanced or metastatic pancreatic endocrine carcinoma patients, chromogranin A (CgA) levels were assessed at baseline and within 4 months of first cycle fluorouracil, doxorubicin and streptozocin treatment. A decrease of 30 % in the level of CgA from baseline was found to be significantly correlated to RECIST defined response (*p* = 0.04) [61]. Nucleosomes, neuron-specific enolase (NSE), progastrin-releasing peptide (ProGRP), cytokeratin-19 fragments (CYFRA 21–1) and CEA levels were also investigated in a study of 128 small cell lung cancer patients treated with various first line chemotherapy regimens (eg. carboplatin, etoposide, and vincristine) to assess response. Patients that responded to therapy had a reduction in these biomarkers [62].

While tumor markers have been used to monitor the effects of systemic therapy for specific tumor types, their general use in oncology practice is hampered by difficulties in interpreting changes [63]. One exception is prostate specific antigen (PSA), which is useful for monitoring treatment effects for prostate cancer. In 118 metastatic CRPC patients treated with next generation androgen pathway inhibitors, a PSA response (>50 % decrease in PSA levels from baseline) at 28 days after treatment initiation was associated with longer PFS and OS [64]. In a group of patients treated with the oral androgen receptor antagonist MDV3100, the model most predictive of prolonged PFS consisted of a prolonged decrease in monthly PSA levels at 12 weeks in conjunction with a reduction in CTCs [65]. Therefore, PSA measurements have found some use in monitoring treatment response in prostate cancer. On the other hand, PSA levels have limited usefulness in bone disease and when cytostatic agents are administered [66–68], as well as when dealing with certain subgroups of prostate cancers that do not produce PSA [69].

#### Tissue-based biomarkers

Direct examination of tumor to evaluate the proliferation marker Ki67 before and after treatment has been used to assess response [70–72]. Following hormonal therapy for breast cancer, a lower Ki67 expression in the surgical specimen was associated with improved survivals [73]. Subsequently, post-treatment Ki67 levels were used as a secondary endpoint in a trial comparing three aromatase inhibitors [5]. While tissue-based biomarkers are less convenient than blood-based biomarkers, there may be some utility in the context of tumors treated with neoadjuvant chemotherapy followed by surgery.

### Developing improved biomarkers of response

The potential benefits for a response biomarker are substantial (Table 1). However, the variable methods for assessing response reflect the need for alternatives. Currently, radiographic techniques are the gold standard for assessing response. However, standard CT and MRI do not always provide a clear signal of response, response may not appear until a drug has been administered for a number of months, and the clinical significance of stable disease is not clear. Functional imaging is intriguing, but imaging methods for assessing response are expensive and inconvenient. Biopsy-based methods are challenging in many situations where tissue samples are hard to access, and they are less attractive as whole because they are invasive. Blood-based biomarkers are perhaps the most intriguing methods under development because they are convenient and much less expensive than radiographic tests.

**Table 1:** Potential benefits of response biomarkers

Benefits to the Patient	Effects on Clinical Practice	Socioeconomic Benefits	Benefits to Industry
Minimal exposure to potentially toxic drugs that are unbeneficial.	Can tailor therapy for patients by development of a biomarker that reflects chemosensitivity and resistance.	Payors (including insurance companies and patients) will pay much less for ineffective drugs.	Clinical trial design would be revolutionized: a) Will provide a new trial endpoint for phase I trials, enabling identification of appropriate doses and patient populations with less harm to trial participants. b) Phase II trials can be performed more quickly, using the biomarker as a surrogate marker for benefit. c) Would greatly facilitate a “go-no go” phase II-III adaptive designs106.
Reduced cumulative toxicities will improve quality of life.	The current practice is to administer a drug until toxicities or disease progression occur. A response biomarker may inform on early chemoresistance. This has the following benefits: a) Inappropriate dose escalations can be avoided. b) Inappropriately prolonged treatments can be avoided. c) Possibility of rotating to a new potentially effective drug regime before progression and clinical deterioration occur.	Patients whose quality of life is preserved and whose disease is controlled with less toxicity will be more likely to be able to resume normal work activities.	Subpopulations that will benefit from drugs will be more easily identified.
Preservation of performance status will facilitate administration of later lines of therapy.	May enable dose titration: lowest effective dose for an individual could be administered.	Novel drug development will be less expensive and more efficient. This may translate to development of more, less costly drugs.	It may become cost effective to screen agents for use in rare cancers.
A response biomarker may expand the therapeutic armamentarium available for patients: low cost trials of drugs on individuals.	A serum biomarker of response would enhance treatment of patients with malignant conditions that are difficult to gauge radiologically e.g. peritoneal disease, bile duct cancer and esophagogastric cancer.		There may be less need for predictive biomarkers, which are specific to each drug, and which take years to develop and validate.

The characteristics of the ideal response biomarker are summarized in Table 2. To identify such a biomarker, we propose a purposeful hypothesis-based approach to discovery and validation. For example, one might devise a biomarker that reflects the presence of tumor based on one of the biological hallmarks of cancer (angiogenesis, inflammation, disordered metabolism, etc.), and a therapeutic response may be manifested as a disappearance of that signal. Alternatively, a biomarker that reflects cell death or a reduction in cell proliferation could be evaluated.

**Table 2:** Characteristics of the ideal response biomarker

Sufficiently sensitive to detect even minor responses that induce disease stabilization.
Specific. Its absence accurately reflects chemoresistance.
Appears rapidly as a result of a response to therapy.
Agnostic to class of antineoplastic drugs.
Applicable to all tumor types.
Easy to measure, amenable to high-throughput testing.
Inexpensive.
Measurement is convenient to the patient and physician.

**Fig. 2:**
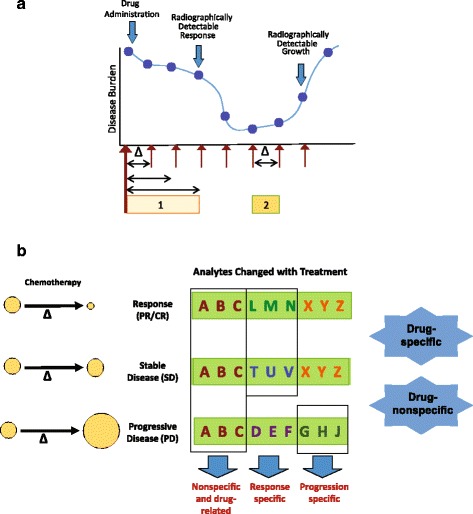
A framework for response biomarker discovery. (A) Serial collection of any biofluid during the course of treatment. Data derived from this experimental design will demonstrate treatment-related changes in biofluids, which can be correlated with response and progression. Data will also be derived that will inform on the biomarker kinetics, including how soon changes occur with response (“1”), as well as how soon changes that indicate acquisition of resistance (“2”) appear. (B) Correlation of treatment-related alterations in biofluids with treatment response. Particularly valuable biomarkers consist of analytes that change specifically with progression (“G,H,J”) or with response (”L, M, N” and possibly “T, U, V”). Iterative experiments related to numerous clinical trials will determine whether these alterations are drugs specific

One experimental framework for discovery would involve the serial collection of blood or urine before and during systemic therapy, correlating changes in those biofluids with radiographic response and progression (Fig. 2). If radiographic response is used as a “gold standard”, then a broad definition of response would be required. For example, RECIST and Choi criteria or PERCIST criteria could be used. In the case of stable disease, to distinguish treatment response from indolent disease, changes associated with prolonged disease-free survival could be identified.

As with any biomarker effort, there will need to be a discovery phase as well as a validation phase. Sufficient numbers of patients will be required to identify the biomarker in the three response categories (partial or complete response; stable disease; and progressive disease). Following identification of the biomarker, a similar approach could be utilized to validate the biomarker in a larger, independent patient cohort.

It is unlikely that a universal biomarker applicable to all therapies (as described above) will emerge in early efforts. Therefore, initial work should focus on response biomarkers that are tumor- and drug-specific. To accomplish this, sufficiently large cohorts receiving the same drugs or drug combinations will be required to identify a response biomarker. Typically, such cohorts would be encountered in a phase III clinical trial. Clinical trials involve a relatively homogeneous population; and outcomes such as response and progression-free survival are well documented following defined treatments. In addition, clinical trials can be utilized to quickly do the discovery experiments, followed by validation experiments. Therefore, clinical trials should be built around this framework of serial sampling before and during therapy.

Once a biomarker is discovered and validated, it will be imperative to understand its kinetics. Does it appear early or late after a response? How long after response is it present? The optimal biomarker will be detectable soon after treatment has been initiated, disappearing with disease progression (or the emergence of chemoresistance).

Ultimately, the biomarker must be reduced to practice. Assay design will have to ensure the reliable and valid measurement of the biomarker. Health economists will help to inform decision makers by demonstrating cost effectiveness of the biomarker compared to standard of care, and also by estimating economic advantages to other stakeholders. Any new biomarker will require prospective assessment of its clinical utility, which will drive uptake in the clinical community. That is, clinicians and policy makers will need to appreciate how the biomarker affects decision-making. Even more dramatic changes to clinical practice would be expected if administration of the new test (and the consequential changes in drug therapy) improved clinical outcomes such as toxicities, quality of life and survival. This will require a randomized controlled trial comparing outcomes in patients treated in the standard fashion (with radiographic and clinical response assessment) and in patients whose response is assessed using the new response biomarker.

## Conclusion

There is a need to individualize cancer therapy, avoiding expensive and toxic drugs that have no benefit. Most of our efforts have been dedicated to identifying predictive biomarkers. While there have been some notable successes using that approach, there remain significant challenges in the identification of predictive biomarkers. The alternative approach is to identify biomarkers that detect response, soon after therapy is initiated, guiding the oncologist to continue or to cease treatment with little exposure to toxic drugs. Despite the significant advantages to that adaptive approach, so far, few efforts have been dedicated to developing response biomarkers. Future efforts should be much more vigorous and purposeful. Reliable and sensitive response biomarkers could potentially revolutionize the way cancer drugs are administered as well as how they are developed.

## Abbreviations

CEA: Carcinoembryonic antigen
CgA: Chromogranin A
CRPC: Castration-resistant prostate cancer
CT: Computed tomography
CTCs: Circulating tumor cells
ctDNA: Circulating tumor DNA
CYFRA 21–1: Cytokeratin-19 fragments
EGFR: Epidermal growth factor receptor
ER: Estrogen receptor
FCH-PET: [18 F] fluorocholine positron emission tomography
FDG: [18 F] fluorodeoxyglucose
FLT-PET: 3′-deoxy-3′-18 F fluorothymidine positron emission tomography
GIST: Gastrointestinal stromal tumors
HCC: Hepatocellular carcinoma
lncRNA: Long non-coding RNAs
miRs: MicroRNAs
MRI: Magnetic resonance imaging
NSE: Neuron-specific enolase
PERCIST: Positron emission tomography response criteria in solid tumors
PET: Positron emission tomography
PFS: Progression free survival
ProGRP: Progastrin-releasing peptide
PSA: Prostate specific antigen
RCC: Renal cell carcinoma
SUV: Standardized uptake value
